# Characteristics of the Immune Cell Infiltration Landscape in Gastric Cancer to Assistant Immunotherapy

**DOI:** 10.3389/fgene.2021.793628

**Published:** 2022-01-06

**Authors:** Chenlu Li, Jingjing Pan, Yinyan Jiang, Yan Yu, Zhenlin Jin, Xupeng Chen

**Affiliations:** ^1^ Department of Gastroenterology, Affiliated Yueqing Hospital, Wenzhou Medical University, Wenzhou, China; ^2^ Department of Laboratory Medicine, The First Affiliated Hospital of Wenzhou Medical University, Wenzhou, China; ^3^ Department of Hematopathology, The First Affiliated Hospital of Wenzhou Medical University, Wenzhou, China; ^4^ Wenzhou Medical University, Wenzhou, China

**Keywords:** gastric cancer, immune cell infiltration landscape, tumor microenvironment, immunotherapy, immune response

## Abstract

**Background:** Gastric cancer (GC) was usually associated with poor prognosis and invalid therapeutical response to immunotherapy due to biological heterogeneity. It is urgent to screen reliable indices especially immunotherapy-associated parameters that can predict the therapeutic responses to immunotherapy of GC patients.

**Methods:** Gene expression profile of 854 GC patients were collected from The Cancer Genome Atlas (TCGA) and Gene Expression Omnibus (GEO) datasets (GSE84433) with their corresponding clinical and somatic mutation data. Based on immune cell infiltration (ICI) levels, molecular clustering classification was performed to identify subtypes and ICI scores in GC patients. After functional enrichment analysis of subtypes, we further explored the correlation between ICI scores and Tumor Mutation Burden (TMB) and the significance in clinical immunotherapy response.

**Results:** Three subtypes were identified based on ICI scores with distinct immunological and prognostic characteristics. The ICI-cluster C, associated with better outcomes, was characterized by significantly higher stromal and immune scores, T lymphocytes infiltration and up-regulation of PD-L1. ICI scores were identified through using principal component analysis (PCA) and the low ICI scores were consistent with the increased TMB and the immune-activating signaling pathways. Contrarily, the high-ICI score cluster was involved in the immunosuppressive pathways, such as TGF-beta, MAPK and WNT signaling pathways, which might be responsible for poor prognosis of GC. External immunotherapy and chemotherapy cohorts validated the patients with lower ICI scores exhibited significant therapeutic responses and clinical benefits.

**Conclusion:** This study elucidated that ICI score could sever as an effective prognostic and predictive indicator for immunotherapy in GC. These findings indicated that the systematic assessment of tumor ICI landscapes and identification of ICI scores have crucial clinical implications and facilitate tailoring optimal immunotherapeutic strategies.

## Introduction

As one of the most common tumors with a high morbidity and mortality, gastric cancer (GC) leads to a poor prognosis and increases critical social burden with 5.7% incidence and 8.2% mortality rates (Bray et al., 2018). More than 50% of diagnosed GC patients were at advanced stages and the prognosis of GC was relatively poor with only less than 30% overall 5-year survival rate (Yang et al., 2020a; Wang et al., 2021). Despite remarkable progress have been made for the treatment of GC, including radiotherapy, chemotherapy and surgery according to different locations and clinical stages, there is still lack of effective strategies for the advanced GC treatment (Ai and Wang, 2020). Recently, the rapid rise of immunotherapy has brought a new therapeutic landscape for the patients who didn’t benefit from conventional chemotherapy, radiation or surgery (Chivu-Economescu et al., 2018). However, in clinical practice, the majority of GC patients were usually still lack of effective therapeutical response to immunotherapy (Li et al., 2020). Therefore, it is crucial to screen reliable index especially immunotherapy-related biological parameters that can predict the therapeutic responses to immunotherapy of GC patients.

Tumor microenvironment (TME) is the inner environment of malignant tumor progression and reveals the biological process of host anti-tumor immune response and destruction of normal tissue. Therefore, the TME was considered as an essential element for exploring the relationship between immune response and tumors with immune cell infiltration (ICI) (Anderson et al., 2006). The TME of tumor tissue was usually complex and associated with tumor initiation, development and prognosis, of which massive immune cells were infiltrated and played great significance to the prognosis of patients (Chen et al., 2020). For instance, tumor-infiltrating lymphocytes (TLS) such as CD4^+^ T cell and CD8^+^ T cell could remarkably improve the curative effects and survival rates (Vassilakopoulou et al., 2016). In addition, tumor-associated macrophages (TAMs), accounting for the majority of leukocytes, had been reported to be involved in the progress of lung and kidney tumors through secreting immunosuppressive cytokines (Vilaseca et al., 2017; Tie et al., 2020). Besides various immune cells, the hypernomic infiltration of stromal components in tumor tissues could decrease the TLS trafficking into tumors (Senbabaoglu et al., 2016). All these researches indicated that intercellular relationships were more significant than the single cell population in TME and the comprehensive landscape of immune cells infiltrating of TME in GC patients still remained unclear.

The identification of potential subtypes of GC by high-throughput technologies may contribute to elucidating the molecular mechanism, improving therapeutic response, and providing insight into any possible evaluating indicators for immunotherapy. In this study, based on the gene expression profile of GC. we applied two major computational algorithms, CIBERSORT and ESTIMATE, to acquire immune clustering subtypes, establish the immune cells infiltration (ICI) scores and further assess the comprehensive landscape about the infiltration of immune cells in GC. Besides, the biological characteristics of ICI subgroups was elucidated and the significance of ICI scores in the prediction of immunotherapy and common chemotherapeutics response was further estimated to validate the ICI landscape for GC.

Conclusively, we are convinced that this study would help in the identification of potential subtypes of GC for interpreting the discriminatory curative responses to immunotherapy and facilitating understanding of the underlying mechanisms of the disease.

## Materials and Methods

### Data Preparation and Preprocessing

The flow chart of our study was showed in Supplementary Figure S1. Transcriptome profiling data of 854 GC samples with their corresponding clinical and mutation data were downloaded from two publicly available datasets, of which the RNA-seq transcriptome data of 407 GC patients with fragments per-kilobase million (FPKM) value were derived from The Cancer Genome Atlas (TCGA) datasets (https://portal.gdc.cancer.gov/) and other microarray data of 447 GC patients (GSE84433) were derived from the Gene Expression Omnibus (GEO) datasets (https://www.ncbi.nlm.nih.gov/geo/). To standardize the expression levels between different sequencing technologies, the FPKM value of TCGA-STAD datasets was transformed into the transcripts per-kilobase million (TPMs) form, which was consistent with the microarray datasets (Wagner et al., 2012). In addition, the “ComBat” algorithm of “sva” package was further applied to remove the non-biological technical biases due to batch effects between different datasets (Leek et al., 2012).

### Consensus Cluster Analysis for Immune Cells Infiltration in Gastric Cancer

To evaluate the immune cell infiltration (ICI) characteristics of GC tissues, we used the “CIBERSORT” package (Chen et al., 2018) to quantitatively analyze the infiltration levels of different immune cells with the LM22 signatures by 1,000 random permutations. Immune cell infiltration levels and stromal contents in different samples were evaluated by using the “ESTIMATE” algorithm (Yoshihara et al., 2013). Then, we performed hierarchical clustering analysis according to the immune infiltration of each sample and the “ConsensuClusterPlus” R package (Wilkerson and Hayes, 2010) was applied to conducted unsupervised clustering based on Euclidean distance and Ward’s linkage methods with 1,000 repeated times to ensure the stability of classification. We performed multiple comparisons among different immune-subtypes including tumor microenvironment (TME) and ICI features to explore the immune characteristics. Moreover, R packages “survival” (Therneau and Lumley, 2015) and “survminer” (Kassambara et al., 2017) were used to perform Kaplan-Meier survival analysis and create survival curves between immune subtypes.

### Identification of ICI Gene-Types and Functional Enrichment Analysis

ICI-associated genes were identified among ICI subtypes using the “limma” package (Smyth, 2005) through setting significance cutoff as adjusted *p* < 0.05 and absolute fold-change >1 and those genes were also divided into different clusters using hierarchical clustering. In order to clarify the biological function and characteristics of gene-clusters, Gene Ontology (GO) enrichment analysis was performed by using “ClusterProfiler” package (Yu et al., 2012) and similar comparisons between gene-types were conducted including TME, ICI and survival analysis. In addition, we also compared the difference of TNM stages between ICI clusters through the chi-square test using the “ggstatsplot” R package.

### Definition and Immune Characteristics of ICI Scores

Based on the unsupervised clustering of expression value of ICI-associated genes, those GC samples were redistributed into different gene-clusters and the expression values correlated with clusters were identified as the ICI gene signatures A and B respectively. Moreover, we applied the Boruta algorithm (Kursa and Rudnicki, 2010) to reduce the dimension of above ICI gene signatures and adopted principal component 1 as the signature score by performing the principal component analysis (PCA) (Zhang et al., 2020). Finally, the method similar to Gene expression grade index was applied to define the ICI score as the following formula: ICI score=∑PC1A−∑PC1B . Subsequently, the threshold of ICI scores was identified through the “surv_cutpoint” function of “survival” package and patients were separated into High- and Low-ICI groups. The software of GESA v4.0 was used for gene set enrichment analysis (GSEA) of ICI scores in gastric cancer. To estimate the discriminative capacity of ICI scores in predicting the prognosis of GCs, the “timeROC” package was applied to draw time-dependent receiver operating characteristic (ROC) curves (Blanche and Blanche, 2019).

### Calculation and Analysis of Tumor Mutation Burden

To explore the relationship between TMB and ICI score, we also downloaded the mutation data of GC patients from TCGA datasets and calculated TMB scores by using the “maftool” R package (Mayakonda et al., 2018). In addition, the correlation analysis between TMB and ICI scores was performed based on Spearman correlation coefficients and combined survival analysis was further employed to clarify the prognostic value. Furthermore, comprehensive mutation analysis was conducted by “maftools” package and mutational signatures of the top 20 genes were further chosen subsequent comparison between ICI-score subgroups using chi-square test.

### Exploration of the Significance of ICI Scores in Clinical Immunotherapy Response

Another independent dataset, IMvigor210, included 298 urothelial cancer patients receiving anti-PD-L1 immunotherapy with complete clinical information and was downloaded from the freely available “IMvigor210CoreBiologies” package (http://research-pub.gene.com/IMvigor210CoreBiologies). Moreover, to evaluate the therapeutic value of ICI scores in the clinic for GC treatment, we calculated the half maximal inhibitory concentration (IC50) of common chemotherapeutic drugs based on Genomics of Drug Sensitivity in Cancer (GDSC) databases (Yang et al., 2013). From the GDSC database, Antitumor drugs such as 5-Fluorouracil, Bleomycin, Cisplatin, Docetaxel and Mitomycin-C have been recommended for the GC treatment by current clinical guidelines. Difference of IC50 of these chemotherapeutic drugs between ICI-score subgroups was compared using Wilcoxon test and the results were exhibited in box diagrams using the “ggpubr” package (Whitehead et al., 2019).

## Results

### The Landscape of Immuno-Cell Infiltration in the TME of Gastric Cancer

**FIGURE 1:**
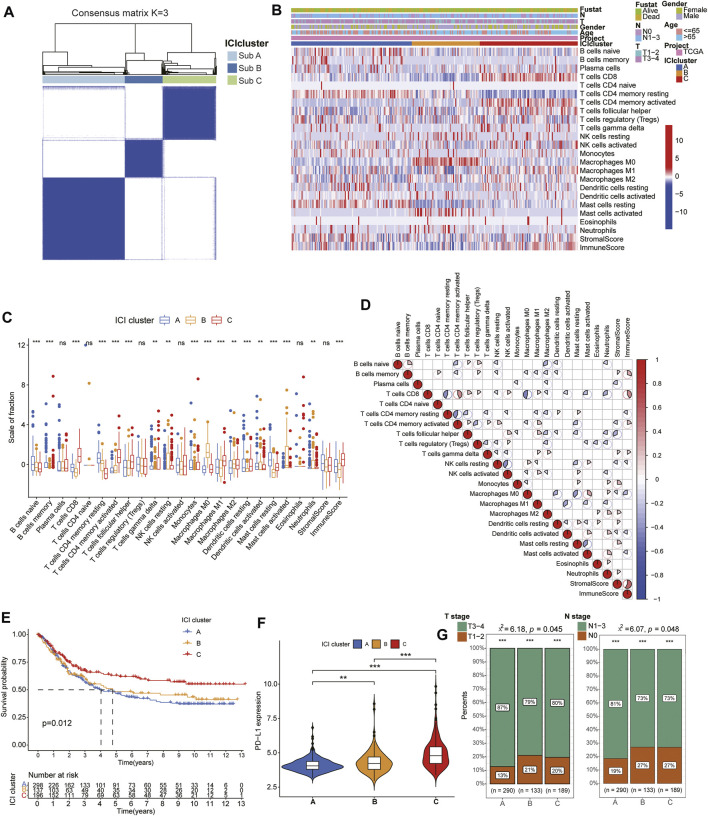
Identification of immune molecular subtypes and characteristics of immuno-cell infiltration landscape in the gastric cancer. **(A)** Consensus clustering matrix for k = 3 in GC patients. **(B)** Heatmap of immune cells infiltration and clinicopathologic features of the three subtypes. **(C)** The box plots showing the difference of immune cells infiltration among three ICI clusters. **(D)** The correlation among the immune cell infiltration in GC patients. **(E)**. Kaplan-Meier curves of overall survival (OS) for the GC patients in three subtypes. **(F)** The expression of PD-L1 between different ICI cluster groups. **(G)** Difference of TMN stages among different ICI cluster groups.

First, the “CIBERSORT” and “ESTIMATE” algorithm were used to calculate the activity or enrichment levels of immune cells in GC tumor tissues (Supplementary Table S1,2). Unsupervised clustering was applied to classify the GC patients into distinct subtypes by the “ConsesusClusterPlus” package based on 854 tumor samples with matched immune cell infiltration (ICI) profiles from the databases (GSE84433 and TCGA-GC). Three independent ICI subtypes had been identified with significant survival differences (log rank test, *p* = 0.012) and ICI analysis revealed complicated relation among immune cells in the TME of GC tissues (Figure 1A,D). In order to further examine the relationship of intrinsic biological differences and distinct clinical phenotypes, we compared the composition of immune cells in TME according to the three ICI subtypes. Among the three subtypes, the ICI cluster C exhibited the longer median survival time (Figure 1E) and higher infiltration of T lymphocytes including CD8^+^ T cells, activated memory CD4^+^ T cells, follicular helper T (Tfh) cells, M1 macrophages and resting dendritic cells (DCs) (Figure 1B,C). With a median survival of 4 years, the ICI cluster A had the worst prognosis and it was characterized by high infiltration of naive B cells, resting memory CD4^+^ T cells, activated DCs and resting Mast cells. The ICI cluster B was marked by high infiltration of M0 and M1 macrophages with about 4.8 years’ median survival time. Moreover, we also analyzed the expression of significant immune checkpoint, PD-L1, in each ICI cluster to estimate the response to immunotherapy. Consistent with the results of survival analysis, the expression of PD-L1 was higher in ICI cluster C than that in ICI cluster A and B (Figure 1F). In addition, the comparison of TNM stages showed that Cluster A displayed more proportion of severe stages than that of Cluster B and C (Figure 1G).

### Identification of ICI Gene-Types and its Functional Enrichment

**FIGURE 2:**
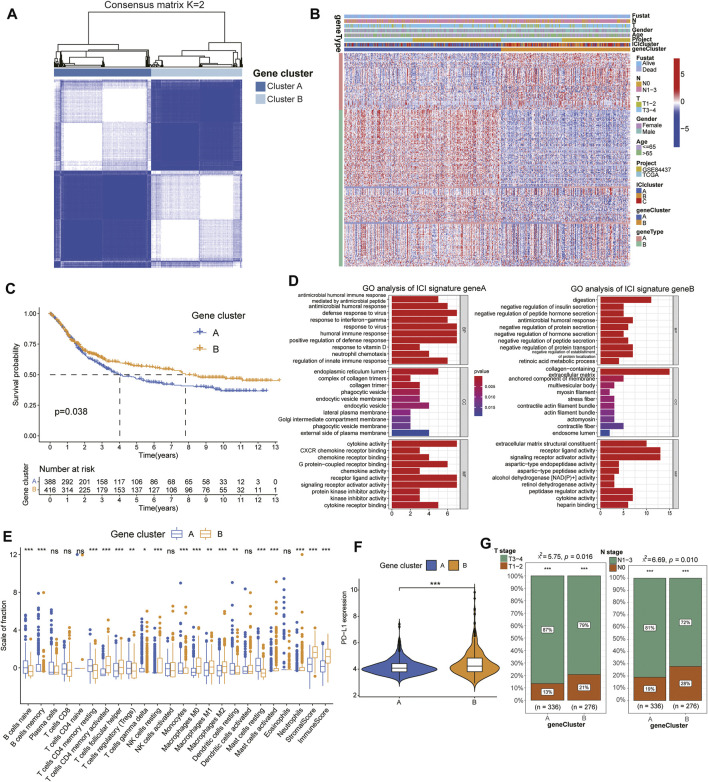
Identification of ICI gene-types and its functional enrichment. **(A)** Consensus clustering matrix for k = 2 in GC patients based on the expression of DEGs. **(B)** Unsupervised clustering of DEGs to classify GC patients into novel two gene clusters **(A,B)**. **(C)** Kaplan-Meier curves for the two gene clusters of patients. **(D)** Gene Ontology enrichment analysis of the two ICI-related signature genes. **(E)** The difference of immune cells infiltrating in TME between two gene clusters. **(F)** The expression of PD-L1 between different gene cluster groups. **(G)** Difference of TMN stages between different gene cluster groups.

To further elucidate the underlying biological characteristics of different immunophenotypes, the differential transcriptome variations analysis was performed among subtypes using the “limma” package. Subsequently, we reperformed the unsupervised hierarchical clustering based on the expression of 251 differentially expressed genes (DEGs) and classified the GC cohorts into two genomic clusters named gene clusters A and B (Figure 2A, Supplementary Table S4). Moreover, those DEGs were positively/negatively associated with ICI signatures and also classified into two clusters: ICI signature gene A and B (Figure 2B) and survival analysis exhibited gene clusters A had a longer median survival time than cluster B (log rank test, *p* = 0.038, Figure 2C). Functional enrichment analysis revealed the ICI signature gene A was significantly enriched in the process of humoral immune response, such as antimicrobial humoral response, defense response to virus and response to interferon-gamma, while the ICI signature gene B was associated with the regulation of digestion, including negative regulation of insulin secretion, peptide hormone secretion and protein secretion (Figure 2D, Supplementary Table S5). In addition, in order to explore the prognostic implications of the ICI gene clusters, we also performed the Kaplan-Meier survival analysis and the gene cluster B had a better prognosis than cluster A (Figure 2C). Interestingly, TME analysis indicated gene cluster B had higher infiltration of immune cells and were associated with significantly high immune scores, suggesting its pro-tumor or anti-tumor activity (Figure 2E). Additionally, the two genomic clusters also showed significant differences in the expression of PD-L1 and the gene cluster B exhibited higher PD-L1 levels (Figure 2F). Consistent with the results of survival analysis, cluster A exhibited more severe TNM features than that of cluster B, suggesting the latter cluster might have a better prognosis and efficacious curative responses (Figure 2G). All these results indicated the consistency between the ICI analysis and prognostic profile in different gene clusters suggesting the scientificity and rationality of our classification method.

### Construction and Identification of Characteristics for ICI Score

**FIGURE 3:**
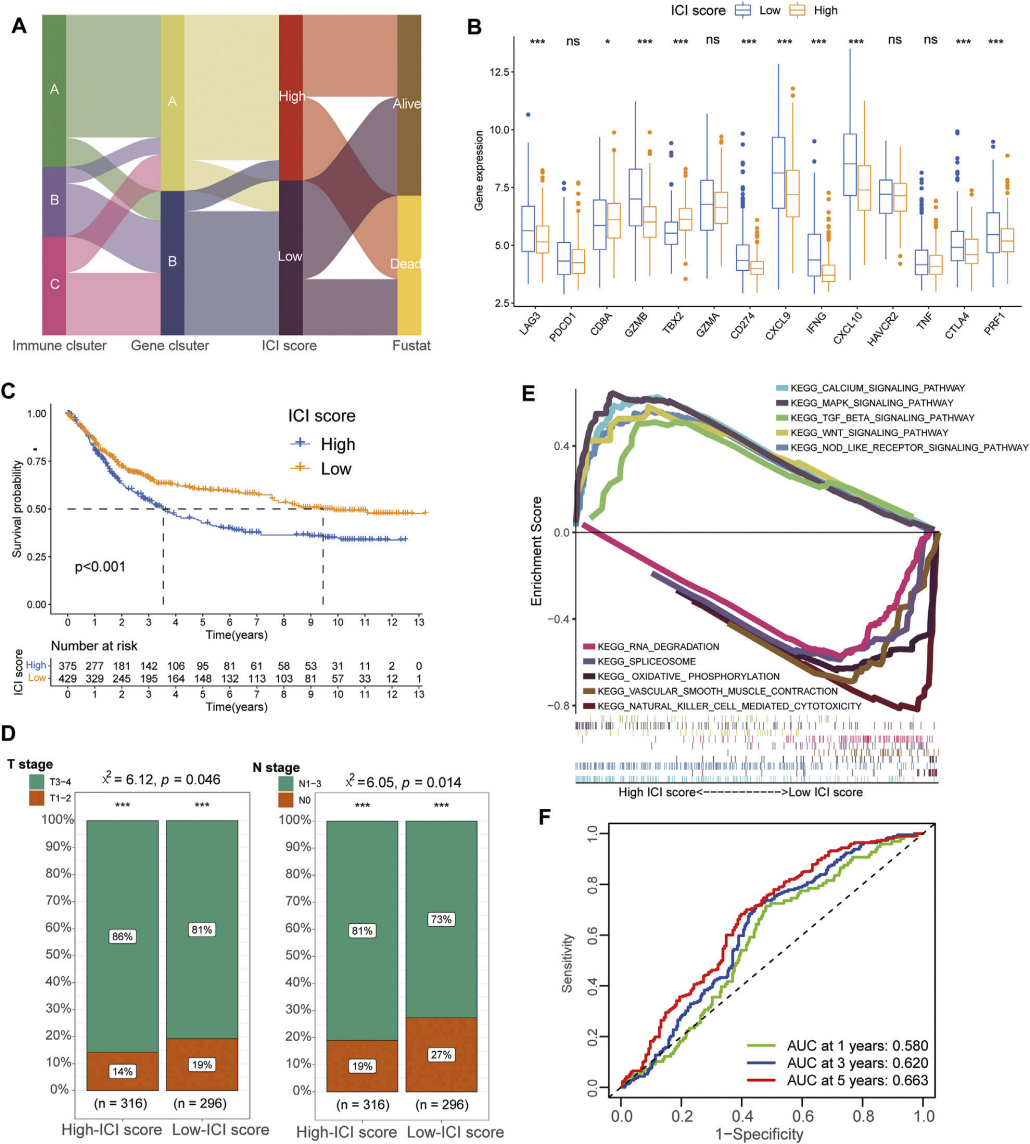
Construction and identification of characteristics for ICI Score. **(A)** Alluvial diagram showing the ICI gene cluster distribution from different ICI gene clusters, ICI score groups and final survival outcomes. **(B)** The expression of immune-checkpoint-associated signatures (CD274/PD-L1, PDCD1, LAG3, CTLA4 and HAVCR2) and immune-activity-related genes (CD8A, CXCL9, CXCL10, GZMA, GZMB, PRF1, IFNG, TNF and TBX2) in different ICI score groups. **(C)** Kaplan-Meier curves of overall survival (OS) for the GC patients in high and low ICI score groups. **(D)** Difference of TMN stages between different ICI score groups. **(E)** The results of GSEA showing that Calcium signaling pathway, MAPK signaling pathway, TGF beta signaling pathway, WNT signaling pathway and NOD like receptor signaling pathway were significantly enriched in high-ICI score group while RNA degradation, Spliceosome, Oxidative phosphorylation, Vascular smooth muscle contraction and Natural killer cell mediated cytotoxicity were enriched in the low-ICI score group. **(F)** ROC analysis showed the 1-year, 3-year, and 5-year AUC values of the ICI scores in predicting the prognosis of GCs were 0.580, 0.620, and 0.663, respectively.

To acquire quantitative index of ICI landscape in GC, we defined ICI scores using principal component analysis and successfully divided the patients into High- and Low-ICI score subgroups (Supplementary Table S7). Figure 3A showed the distribution procedure of different subgroups and the gene cluster A was almost divided into High-ICI score cohorts while massive cluster B was contributed into Low-ICI score subgroups. Furthermore, we also evaluated the immune activity and immune tolerance condition of each cohort before determining the prognostic value of ICI scores. To accomplish the evaluation, immune-checkpoint-associated signatures were chosen to assess the response of immunotherapy including CD274/PD-L1, PDCD1, LAG3, CTLA4 and HAVCR2 while immune-activity-related genes were selected to estimate the condition of immune activation including CD8A, CXCL9, CXCL10, GZMA, GZMB, PRF1, IFNG, TNF and TBX2. We observed that most signatures of immune-checkpoint and immune-activity-relevant genes were significantly upregulated in the Low-ICI score groups except PDCD1, CD8A, HAVCR2, TBX2 and TNF (Figure 3B) and Low-ICI group also had a better prognosis than High-ICI score cohorts (Figure 3C). Clinical analysis of TNM stages also demonstrated that Low-ICI scores subgroups exhibited more frequent phenotypes with high-levels of clinical stages (Figure 3D). Moreover, GSEA analysis also revealed that Calcium signaling pathway, MAPK signaling pathway, TGF beta signaling pathway, WNT signaling pathway and NOD like receptor signaling pathway were significantly enriched in high-ICI score group while RNA degradation, Spliceosome, Oxidative phosphorylation, Vascular smooth muscle contraction and Natural killer cell mediated cytotoxicity were enriched in the low-ICI score group (Figure 3E, Supplementary Table S6). Moreover, time-dependent ROC analysis showed the 1-year, 3-year, and 5-year AUC values of the ICI scores in predicting the prognosis of GCs were 0.580, 0.620 and 0.663, respectively (Figure 3F).

### The Relationship Between ICI Scores and Tumor Burden Mutation

**FIGURE 4:**
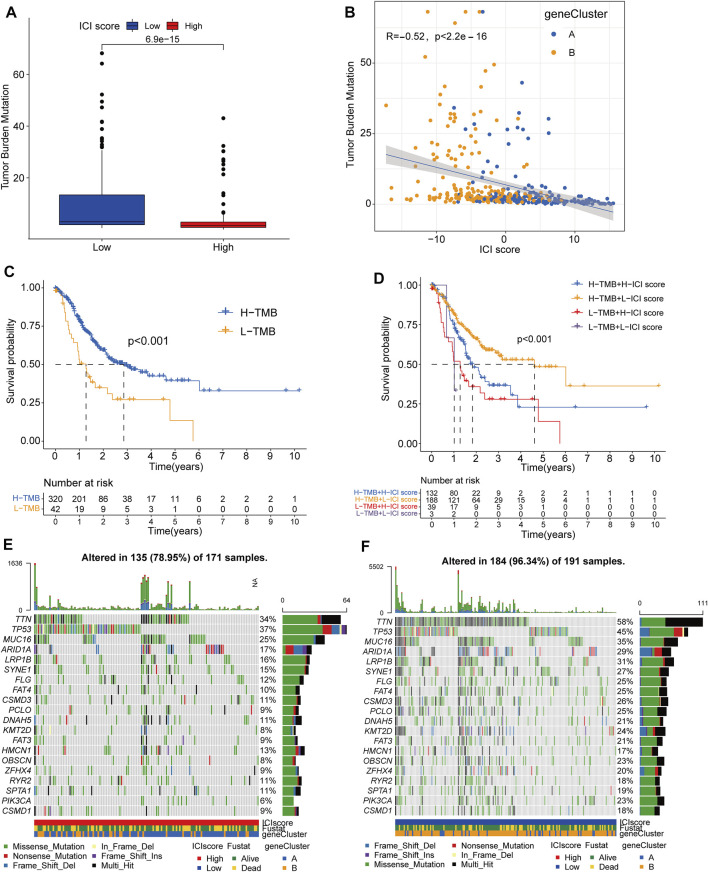
The Relationship between ICI Scores and Tumor Burden Mutation. **(A)** The difference of TMB value between the high and low ICI score subgroups. **(B)** The scatter diagram showing the negative correlation between TMB value and ICI scores. **(C)** Kaplan-Meier curves of the high and low TMB subgroups in GC patients. **(D)** Stratified survival analysis for GC patients combining TMB groups and ICI score subtypes. **(E,F)** The oncoPrint showing the mutant situation of individual patients in high ICI scores groups (red) and low ICI scores groups (blue) respectively.

Increasing evidence indicated that tumor burden mutation (TMB) could affect the infiltration of CD8^+^ T cells, which was considered as significant elements in alleviating the prognosis of tumors (Rizvi et al., 2015; Cristescu et al., 2018). These studies implied that TMB might determine the individual’s response to target immunotherapy. To investigate the intrinsic relationship between TMB and ICI scores, we compared the levels of TMB scores between ICI score subgroups and performed Spearman correlation analysis. The results revealed high-ICI score group had a lower TMB scores than that of low-ICI score cohorts (Wilcox test, *p* < 0.05) and the ICI scores were negatively correlated with TMB scores (Spearman coefficient: R = −0.52, *p* < 2.2e-16) (Figure 4A,B). Longer median survival time was also identified in high-TMB groups than low-TMB subgroups by survival analysis, consistent with the prognosis of low-ICI score groups (Figure 4C). Moreover, the stratified survival analysis further revealed patients with high TMB and low ICI scores had the best prognosis status, suggesting the synergistic effect of TMB and ICI scores in prognostic stratification of GC. Besides, low-ICI score cohorts still exhibited a better prognosis than that of high-ICI groups even in GC patients with same TMB stages and in patients with same ICI-score condition, high-TMB patients also had a longer median survival time than low-TMB cohorts (Figure 4D). These results implied the negative correlation between ICI scores and TMB values and their potential complementary value in the application of prognosis for GC patients.

Furthermore, we estimated the distribution of somatic variants between the low and high ICI subgroups based on the TCGA datasets. The results revealed various mutation patterns were identified in both high- and low-ICI subgroups including Missense Mutation, Nonsense Mutation, Frame Shift Del and In Frame Del, and more frequent mutations were observed in low-ICI groups (96.34%) than that of high-ICI groups (78.95%). The top20 genes with most mutation frequency were exhibited in Figure 4E,F, of which TTN, PIK3CA, KMT2D and OBSCN were significantly different between the low and high ICI score groups (chi-square test; *p* < 0.05) and the top20 genes with significantly difference were displayed in Table1. These results might propose novel ideas for exploring the potential mechanism of tumor ICI composition and gene mutation in immune checkpoint therapy.

**TABLE 1:** Top20 Somatic Variants between High- and Low-ICI Score group.

Gene symbol	High ICI score (%)	Low ICI score (%)	*p* Value
TTN	58 (33.92%)	111 (58.12%)	6.75E-06
PLEC	7 (4.09%)	39 (20.42%)	6.86E-06
CNTLN	3 (1.75%)	29 (15.18%)	1.65E-05
PIK3CA	11 (6.43%)	43 (22.51%)	3.48E-05
ANKRD11	4 (2.34%)	29 (15.18%)	5.00E-05
HDAC4	0 (0%)	19 (9.95%)	6.31E-05
KMT2D	13 (7.6%)	45 (23.56%)	6.64E-05
ANK3	8 (4.68%)	36 (18.85%)	7.56E-05
ASPM	4 (2.34%)	28 (14.66%)	8.25E-05
HERC2	6 (3.51%)	32 (16.75%)	8.40E-05
JARID2	1 (0.58%)	21 (10.99%)	8.91E-05
NPAP1	1 (0.58%)	21 (10.99%)	8.91E-05
SIPA1L1	2 (1.17%)	23 (12.04%)	1.11E-04
SLITRK5	4 (2.34%)	27 (14.14%)	1.35E-04
FBN1	4 (2.34%)	27 (14.14%)	1.35E-04
SSPO	4 (2.34%)	27 (14.14%)	1.35E-04
HIVEP1	1 (0.58%)	20 (10.47%)	1.49E-04
OBSCN	13 (7.6%)	43 (22.51%)	1.63E-04
KMT2A	4 (2.34%)	26 (13.61%)	2.21E-04
ATP10 A	4 (2.34%)	26 (13.61%)	2.21E-04

*p* value was obtained based on the chi-square test between the high and low ICI, score subgroups.

The significance of ICI scores in the prediction of immunotherapy and common chemotherapeutics response.

**FIGURE 5:**
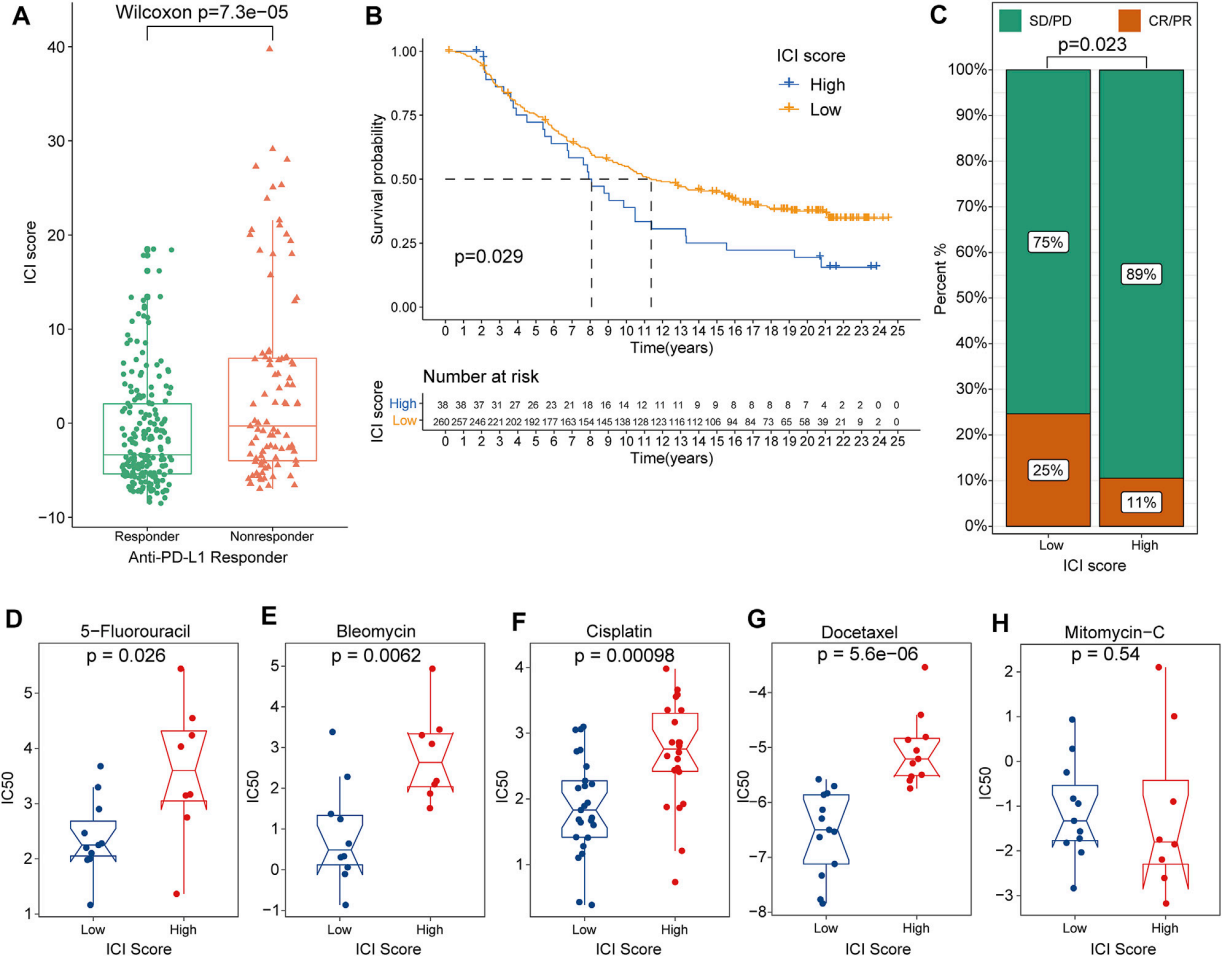
The role of ICI scores in the prediction of immunotherapy and common chemotherapeutics response. **(A)** ICI scores between groups with different clinical immunotherapy response status. **(B)** Survival analysis for patients in high and low ICI score groups from the IMvigor210 cohort. **(C)** The distribution of the complete remission (CR)/partial response (PR) rate and stable disease (SD)/progressive disease (PD) to anti-PD-L1 immunotherapy between high and low ICI score groups based on the IMvigor210 cohort. **(D–H)** The difference of IC50 value from five common chemotherapy drugs between high and low ICI score groups, including 5-Fluorouracil, Bleomycin, Cisplatin, Docetaxel and Mitomycin-C.

To further explore the role of ICI scores in predicting the therapeutic benefit in GC, the patients who accepted anti-PD-L1 immunotherapy from the IMvigor210 cohort were calculated ICI scores and assigned into high- or low-ICI scores groups (Supplementary Table S8). Notably, the effective response rate of anti-PD-L1 therapy was significantly higher in the low-ICI score group than in high-ICI subgroups and the low-ICI patients outlived the high-ICI score patients (Figure 5A,B). Moreover, the rate of complete remission (CR) after immunotherapy was also increased in low ICI scores than high ICI cohorts (Figure 5C).

Besides checkpoint blockers therapy, we also attempted to investigate the potential associations between ICI scores and the curative efficacy of common chemotherapeutics in treating gastric cancers. IC50 was calculated and five common anti-GC chemotherapeutic drugs were obtained from the GDSC databases including 5-Fluorouracil, Bleomycin, Cisplatin, Docetaxel and Mitomycin-C (Supplementary Table S9). Interestingly, except Mitomycin-C, other four drugs all exhibited lower IC50 value in low-ICI score groups indicating the low-ICI patients might obtain better curative efficacy from common chemotherapy (Figures 5D–H). Collectively, these outcomes indicated that ICI scores could be associated with the response to immunotherapy and common chemotherapy.

## Discussion

As a malignant tumor with high mortality, the prognosis of GC remains poor without effective therapeutical tools. Despite the development of combination chemotherapy, consisting of platinum and 5-fluorouracil, only a mild survival advantage was obtained in GC patients (Galdy et al., 2016). Recently, cancer target immunotherapies have acquired considerable attention as an effective and accurate therapeutic option for GC including immune checkpoint inhibitors, tumor vaccines and chimeric antigen rector (CAR)-T cells (Yang et al., 2019). However, even if the GC patients were at the same clinical stage, their prognosis and therapeutical response to the same treatment might be still different in clinical practice. Gullo’s study has also reported this phenomenon and attributed to genomic and biological heterogeneity (Gullo et al., 2018). Therefore, identification of a novel subtype and reliable index to evaluate and predict the therapeutical response to immunotherapy for GS is urgently needed.

In this study, we first proposed an immune molecular subtype based on clustering immune infiltration scores with distinct clinical and immunological signatures in the meta-cohort of 854 GC patients. Interestingly, the characteristics of the three molecular subtypes manifested significant homogeneity. TME analysis revealed higher stromal and immune scores were found in ICI cluster C than other two clusters, indicating anti-tumor immune response was significantly activated in ICI cluster C of GC (Zeng et al., 2019). Moreover, higher infiltration scores of T cells, especially activated CD4^+^ memory T cells, CD8^+^ T cells and follicular helper T cells, which have been regarded as the major immune cells for anti-tumor efficacy (Biase et al., 20192019), were demonstrated in the ICI cluster C and this subtype also presented longer median survival time than other two clusters through Kaplan-Meier survival analysis (Figure 1C,E). Immune checkpoint genes, especially PD-L1, have been demonstrated playing significant role in immune suppression in multiple tumors and the target inhibitors have also been widely applied to immunotherapy for cancers (Kim et al., 2020a). It was worth mentioning that the expression levels of PD-L1 was significantly increased in ICI cluster C subgroups suggesting a higher level of immune exhaustion and potential better therapeutical response in GC patients.

To further explore the potential biological functional features of the ICI subtypes in GC, we fetched the differential expression genes among three subtypes and identified novel ICI gene types. Interestingly, the ICI gene cluster B exhibited a better prognosis for GC than gene cluster A and was positively associated with the expression of ICI signature A, which were significantly enriched in the process of humoral immune response. Conversely, the ICI gene cluster A was positively associated with the ICI signature B and major enriched in the negative regulation of digestion. Increasing evidence had indicated that the *H. pylori* bacteria could actively dampen the T-helper 1 (Th1) response and inhibit CD4/CD8 positive T cell activation and IFN-*γ* production, leading to considerable tissue damage during the progression of GC (Wen et al., 2004; Ito et al., 2008). Therefore, the process of humoral immune response would ameliorate the disease condition and improve the survival for GS patients, interpreting the better prognosis of ICI gene cluster B in our study (Kurtenkov et al., 2007). In addition, we also observed ICI gene cluster B had the higher stromal scores, immune scores, expression levels of PD-L1, milder TNM stages and other immune-response-related cells infiltration, consistent with the results of survival analysis and GO functional enrichment analysis. These outcomes suggested the ICI gene cluster B was associated with the immune-activation condition with better therapeutic reaction and prognosis for GC (Panda et al., 2018).

Considering the individual biological heterogeneity to immune checkpoint inhibitors, it was urgently required to understand the ICI landscape of individual tumors.

In previous studies, tumor subtype-specific biomarkers had been successfully established to improve individual outcome prediction in breast and colorectal cancers, respectively (Callari et al., 2016; Bramsen et al., 2017). In our study, through the Boruta algorithm, we successfully established the ICI score to quantify the ICI pattern and found most low-ICI score groups were corresponding to the former ICI gene cluster B with a longer lifetime. Moreover, the expression levels of most immune-checkpoint-associated and immune-activity-related genes were both significantly increased in the low-ICI score groups, implying the activation of anti-tumor process in gastric cancers. ROC analysis further demonstrated that ICI scores had a good prediction capacity in all 1-year, 3-year and 5-year overall survival for GC patients, indicating the potential predicted value of ICI scores. In addition, GSEA revealed that the genes of high-ICI score cluster were involved in the immunosuppressive pathways, such as TGF-beta, MAPK and WNT signaling pathways, which had been reported associated with the progression of GC (Chen et al., 2014; Jia et al., 2017; Yang et al., 2020b). Contrarily, several immune-activated and metabolic-related pathways were found enriched in low-ICI score cohorts including Natural killer cell mediated cytotoxicity and Oxidative phosphorylation. Su et al. (2020) also identified three oxidative phosphorylation genes associated with the clinical prognostic significance in GC and multiple therapeutic technologies had been found to activate NK cells directly or indirectly to improve their killing activity for GC including cytokines, antibodies, immunomodulatory drugs, immune checkpoint blockades and gene therapy (Mimura et al., 2014).

TMB has been recognized as a new biomarker for immune checkpoint treatment in various tumor types and reported to applied in predicting the survival status after immunotherapy in advanced gastric cancer patients (Kim et al., 2020b). Therefore, TMB value was considered as a sensitive index to immunotherapy. In the current study, we also detected that the TMB was significantly increased in patients with low ICI scores. The significantly negative correlation between the TMB value and ICI scores was identified with the correlation coefficient of −0.52. The stratified analysis revealed that the prognosis value of ICI scores was consistent with TMB values and the patients with high-TMB and low-ICI scores exhibited optimal survival condition. Notably, ICI scores could still exhibit significant discriminating capacity in estimating the survival period of GC patients in same TMB conditions using stratified analysis, indicating that ICI scores might provide insights not available from TMB. Recently, the correlation between gene mutations and response or tolerance to immunotherapy had been identified in published reports (George et al., 2017). In our study, more frequent mutations were observed in low-ICI groups and massive mutable genes with significant variant frequency differences were identified, suggesting somatic mutation might participate in the process of immune-subtypes in GC.

Furthermore, to validate the significance of ICI scores in the prediction of immunotherapy, the patients receiving immunotherapy were evaluated based on the IMvigor210 datasets and we found the ICI score was significantly decreased in patients responded to corresponding immunotherapy, suggesting target immunotherapy might be beneficial tool for the patients with low ICI scores. In addition, the low-ICI score groups also demonstrated longer median survival time and higher rate of complete remission (CR) after immunotherapy in clinical trials. Besides immunotherapy, common chemotherapeutic drugs also be demonstrated lower IC50 value in low-ICI score cohorts including 5-Fluorouracil, Bleomycin, Cisplatin and Docetaxel from GDSC database, implying the low-ICI score patients might be more efficacious against these chemotherapeutic drugs. Overall, these findings from external datasets validated the potential benefits in low-ICI scores and indicated ICI scores might play a vital role in predicting the curative responses to common chemotherapy and immune checkpoint therapy.

However, there are still several limitations in our study. For one thing, the high-throughput sequencing datasets for initial analysis were relatively insufficient because it was simply obtained from the public databases. The corresponding results and conclusion remain to be investigated through more external congeneric researches. For another, there are still several concerns need other researches, even clinical practices, to repeatedly confirm and improve, such as the concrete role of ICI scores in predicting the response to immunotherapy, the optimal threshold for the classification ICI scores and so on.

## Conclusion

In conclusion, we comprehensively explored the ICI landscape of GC, providing a clear visual angle of the characteristics in immune molecular subtypes based on clustering immune infiltration scores with distinct clinical and immunological signatures. The distinction in ICI landscapes was found to be associated with the complexity and heterogeneity of tumor treatment. Moreover, we successfully identified and validated the significance of ICI scores in predicting the therapeutic responses to immunotherapy based on clinical trial data from external datasets. The systematic assessment of tumor ICI landscapes and identification of ICI scores have crucial clinical implications and facilitate tailoring optimal immunotherapeutic strategies.

## Data Availability

The original contributions presented in the study are included in the article/Supplementary Material, further inquiries can be directed to the corresponding authors.
