# Supplementary figures and images for: Characteristics of the Immune Cell Infiltration Landscape in Gastric Cancer to Assistant Immunotherapy
Source: Front Genet. 2022 Jan 6;12:793628. doi: 10.3389/fgene.2021.793628 (PMC8770548; doi:10.3389/fgene.2021.793628)

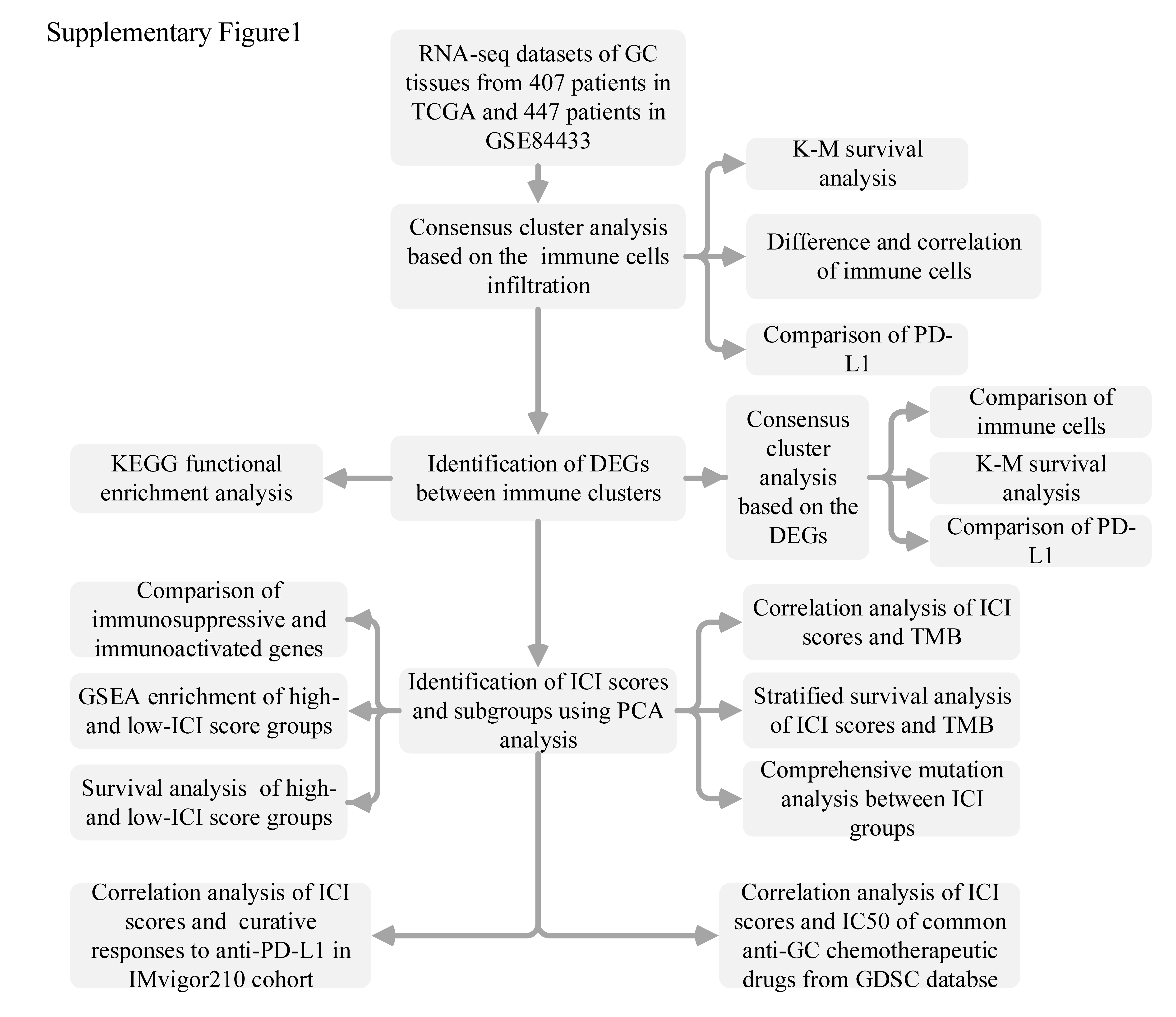

Supplement: Supplementary file 1 [file Image1.TIF]
